# Deciphering pathogenic cellular module at single-cell resolution in checkpoint inhibitor-related pneumonitis

**DOI:** 10.1038/s41388-023-02805-4

**Published:** 2023-08-31

**Authors:** Pengfei Cui, Jinfeng Li, Haitao Tao, Xiaoyan Li, Liangliang Wu, Junxun Ma, Huanhuan Wang, Tingting Liu, Min Zhang, Yi Hu

**Affiliations:** 1 Department of Oncology, the Second Medical Center of Chinese PLA General Hospital, 100853 Beijing, China; 2 Senior Department of Oncology, the Fifth Medical Center of PLA General Hospital, 100853 Beijing, China; 3 Institute of oncology, Senior Department of Oncology, the Fifth Medical Center of PLA General Hospital, 100853 Beijing, China; 4 Institute of oncology, Senior Department of Oncology, the First Medical Center of PLA General Hospital, 100853 Beijing, China; 5 Department of Pulmonary and Critical Care Medicine, the Second Medical Center of Chinese PLA General Hospital, 100853 Beijing, China; 6 State Key Laboratory of Kidney Diseases, Department of Nephrology, the First Medical Center of Chinese PLA General Hospital, 100853 Beijing, China

**Keywords:** Tumour immunology, Cancer microenvironment

## Abstract

Checkpoint inhibitor pneumonitis (CIP) is the most common fatal immune-related adverse event; however, its pathophysiology remains largely unknown. Comprehensively dissecting the key cellular players and molecular pathways associated with CIP pathobiology is critical for precision diagnosis and develop novel therapy strategy of CIP. Herein, we performed a comprehensive single-cell transcriptome analysis to dissect the complexity of the immunological response in the bronchoalveolar lavage fluid (BALF) microenvironment. CIP was characterized by a dramatic accumulation of CXCL13+ T cells and hyperinflammatory CXCL9+ monocytes. T-cell receptor (TCR) analysis revealed that CXCL13+ T cells exhibited hyperexpanded- TCR clonotypes, and pseudotime analysis revealed a potential differentiation trajectory from naïve to cytotoxic effector status. Monocyte trajectories showed that LAMP3+ DCs derived from CXCL9+ monocytes possessed the potential to migrate from tumors to the BALF, whereas the differentiation trajectory to anti-inflammatory macrophages was blocked. Intercellular crosstalk analysis revealed the signaling pathways such as CXCL9/10/11-CXCR3, FASLG-FAS, and IFNGR1/2-IFNG were activated in CIP+ samples. We also proposed a novel immune signature with high diagnostic power to distinguish CIP+ from CIP− samples (AUC = 0.755). Our data highlighted key cellular players, signatures, and interactions involved in CIP pathogenesis.

## Introduction

Immune-checkpoint inhibitors (ICIs) bind to immune-checkpoint proteins to relieve tumor-mediated inhibition and recover T-cell cytotoxic function [1, 2]. ICIs therapy has been proven to be an effective anti-cancer treatment program for a wide range of human malignancies [3, 4]; however, ICIs therapy can cause inflammatory toxicities, also termed immune-related adverse events (irAEs), and even fatal adverse events (FAEs) [5–7]. irAEs can affect any human organ system and induce treatment-limiting toxicities, posing challenges to ICIs use [8–10]. irAEs usually occur at barrier sites, including the gastrointestinal (GI) mucosa, liver, skin, and lung [11, 12], and the FAEs are mainly seen in the respiratory system and are also termed checkpoint inhibitor-associated pneumonitis (CIP) [13, 14]. The incidence of CIP is approximately 3–5% according to clinical trial data [15, 16]; however, the occurrence of CIP can be higher in real-world settings than previously reported [17, 18]. A recent meta-analysis revealed that approximately 35% of anti–PD-1/anti–PD-L1 related fatalities are due to CIP [19]. Therefore, the dynamic assessment of ICIs therapy and effective monitoring of adverse events are important to reduce the incidence and mortality of irAEs and facilitate cancer immunotherapy.

The clinical and pathognomonic radiological features of CIP are nonspecific [20, 21]. Patients with CIP present with an acute-to-subacute onset of dyspnea, hypoxemia, and pulmonary infiltrates, similar to those with acute respiratory distress syndrome [21, 22]. In addition, the current diagnosis of CIP requires the exclusion of other lung injuries, and fundamental knowledge of CIP pathobiology is lacking [23, 24]. Therefore, a comprehensive understanding of CIP pathobiology and the dissection of key cellular players and molecular pathways underlying CIP initiation are critical for cancer immunotherapy, precise diagnosis, and timely prevention of CIP.

Several studies have used flow cytometry and bulk transcriptomic analyses to elucidate the mechanisms underlying CIP [25–27]. However, these studies detected signals at the population level, limiting their ability to capture cellular and molecular heterogeneity and impeding precise diagnosis. Single-cell RNA-sequencing (scRNA-seq) is a powerful technology that widely used to reveal the heterogeneous cellular and molecular characteristics of various cells associated with cancer [28, 29] and inflammatory diseases such as COVID-19 and cancer immunotherapy-induced colitis [30–32].

Herein, we performed scRNA-seq, single-cell T-cell receptor sequencing (scTCR-seq), flow cytometry, and cytokine expression analysis of CIP bronchoalveolar lavage fluid (BALF) and comparatively analyzed the bronchoalveolar immune landscape across ICI-treated non-small cell lung cancer (NSCLC) patients with and without CIP to examine the complexity of the immunological responses in the BALF immune microenvironment of NSCLC patients with CIP.

## Results

### Global analysis of immune cell populations in BALF of patients with CIP

**Fig. 1:**
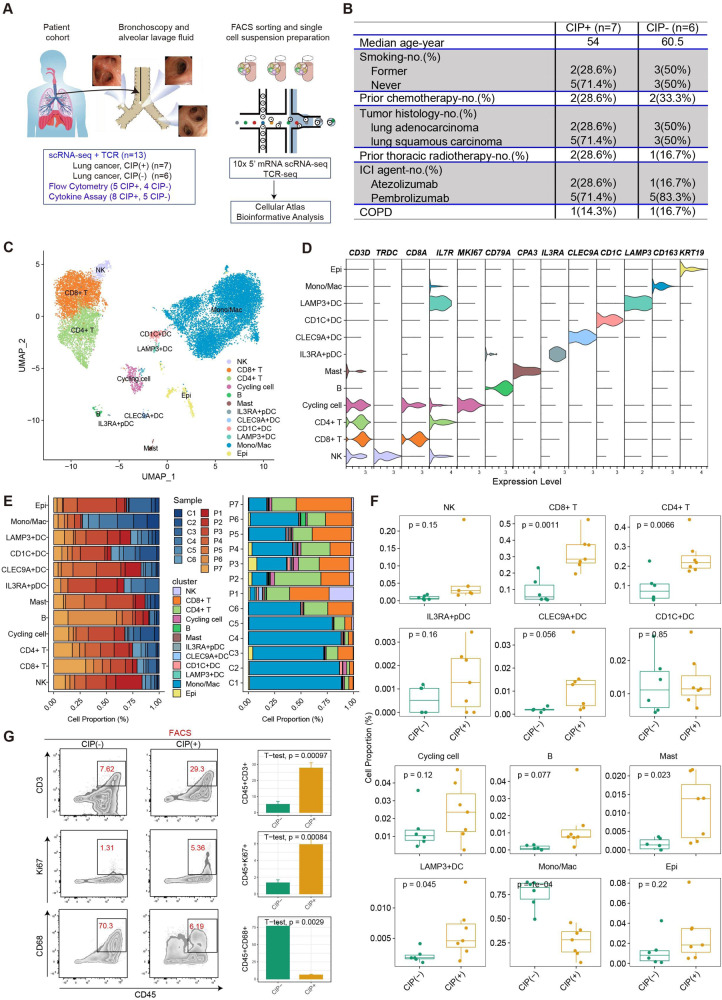
Global analysis of immune cell populations in CIP. **A** A general workflow of BALF collection and processing of single-cell suspensions for scRNA+TCR-seq and flow cytometry. **B** Summary of patient cohorts for scRNA-seq analysis. Unbiased clustering (**C**) and cell type annotation (**D**) of 76,496 high-quality BALF cells. **E** the proportion of sample contributions per annotated cell type (left) and the percentage of annotated cell type contributions per sample (right). **F** Relative contribution of each cell type in distinct pathology samples. *P* values were assessed by Student’s *t* test. **G**, T/NK, Cycling, Mono/Mac frequencies as determined by flow cytometry (percentage of live CD45+ cells), *P* values were assessed by Student’s *t* test.

We present the first in-depth cellular and molecular analysis of immune cell populations in the BALF of patients with CIP induced by ICIs therapy, which enrolled two patient populations from 13 donors: (1) ICI-treated NSCLC patients with CIP (*n* = 7, CIP+), and (2) ICI-treated NSCLC patients without CIP (*n* = 6, CIP−) (Fig. 1A, B). Our study design allowed us to distinguish the key cellular players, molecular pathways, and effector programs induced by ICIs therapy to dissect CIP pathophysiology. All ICI-treated patients had previously been treated with a PD-1/PD-L1 blockade. In the CIP+ group, one patient had grade 1 pneumonitis, six had grade 2 events; four patients had cryptogenic organizing pneumonia (COP) like pneumonitis, two had interstitial pneumonitis, and one had ground-glass opacities (GGO) (Fig. 1B, Supplementary Tables S1–3). Three patients (two in the CIP+ Group and one in the CIP− Group) in this study had received thoracic radiotherapy before ICIs treatment, and no pneumonitis was found in the radiation field. The BALF was obtained immediately after the development of CIP (average, 3.67 days; maximum 8 days) from the newly infiltrated area (Supplementary Table S1). The patients in the CIP+ cohort received corticosteroids after bronchoscopy (Supplementary Table S3). A total of 776,496 high-quality cells passed data quality control (Supplementary Fig. 1A–E), and 25 cell clusters were obtained after integrated bioinformatics analysis, including data integration, normalization, batch effect removal, dimension reduction, and cluster detection. We identified and annotated 12 main cell types according to the expression of canonical gene markers (Supplementary Table S4), which comprised epithelium (SCGB3A1, KRT19), CD8+ T cell (CD3D, CD8A), CD4+ T cell (CD4, IL7R), natural killer (NK) cell (XCL2, TRDC), plasma B cell (MZB1, JCHAIN), B cells (CD79A, CD79B), proliferating cell (MKI67, TOP2A), CD1C dendritic cell (DC) types (CD1C, CLEC9A, LAMP3, IL3RA), monocyte/macrophage (S100A9, APOE, MARCO), and mast cell (CPA3) (Fig. 1C, D, Supplementary Fig. 1F). Each cell subtype contained cells from all the patients, indicating the absence of the main patient-specific batch effects (Fig. 1E). The scRNA-seq findings revealed an increase in the frequency of T/NK and proliferative or cycling cells in the CIP+ group, whereas monocytes/macrophages were depleted (Fig. 1F), which was validated by flow cytometry analysis in another patient cohort (Fig. 1G, CIP+, *n* = 5; CIP−, *n* = 4; Supplementary Tables S5 and 6). These results demonstrate CIP is highly associated with major changes in the immune microenvironment of BALF.

### Characteristics and dynamics of CD8+ T cells in BALF of patients with CIP

**Fig. 2:**
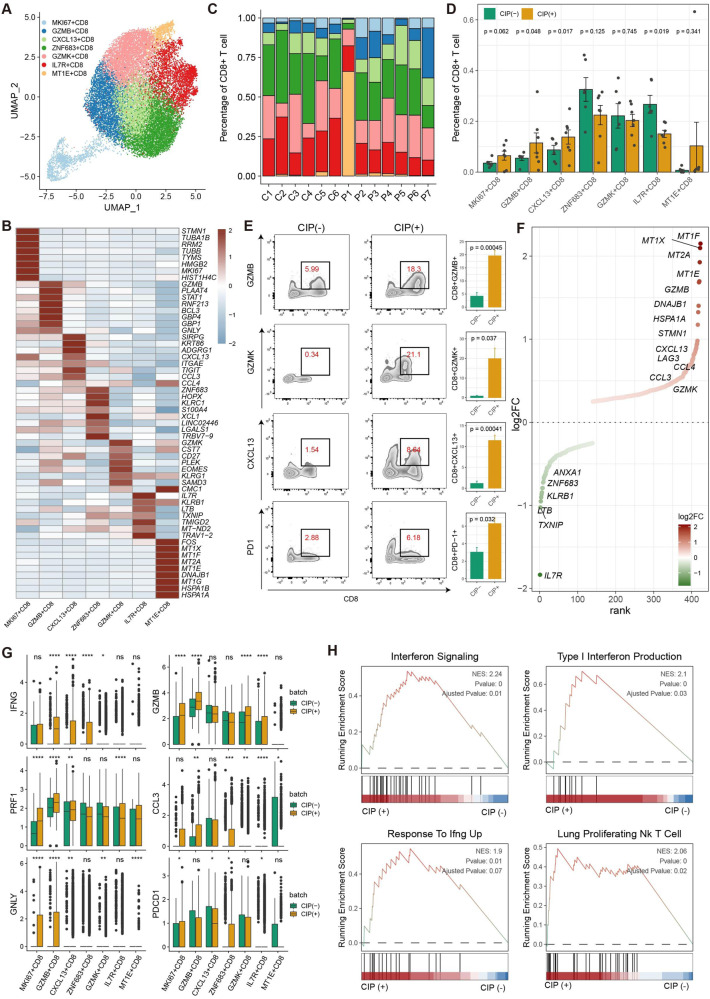
CIP associated CD8+ T Cell with cytotoxic effector programs. **A** Sub-clustering of CD8+ T cells revealed 7 cell-subclusters. **B**, Heat map showing the scaled expression of top 8 differentially expressed genes among eight CD8+ cell-subclusters. **C** The proportion of sample contributions per annotated CD8+ cell-subclusters. **D** Relative contribution of eight CD8+ cell-subclusters in distinct sample pathology. *P* values were assessed by Student’s *t* test. **E** CIP associated CD8+ T frequencies as determined by flow cytometry (percentage of live CD8+ cells), *P* values were assessed by Student’s *t* test. **F** Ranking of significantly differentially expressed genes in CIP associated CD8+ T cell clusters compared with other CD8+ T cell clusters. **G** Box plots showing the expression of immune-checkpoint (PDCD1), immune cell-homing signals (CCL3), effector (IFNG, GNLY) and cytotoxic (GZMB, PRF1) markers in CD8+ cell-subclusters in distinct pathology samples. *P* values were assessed by Wilcoxon test, *P < 0.05, **P < 0.01, ***P < 0.001. **H** GSEA plots showing pathways enriched in CIP associated CD8+ T cell clusters compared with other CD8+ T cell clusters.

**Fig. 3:**
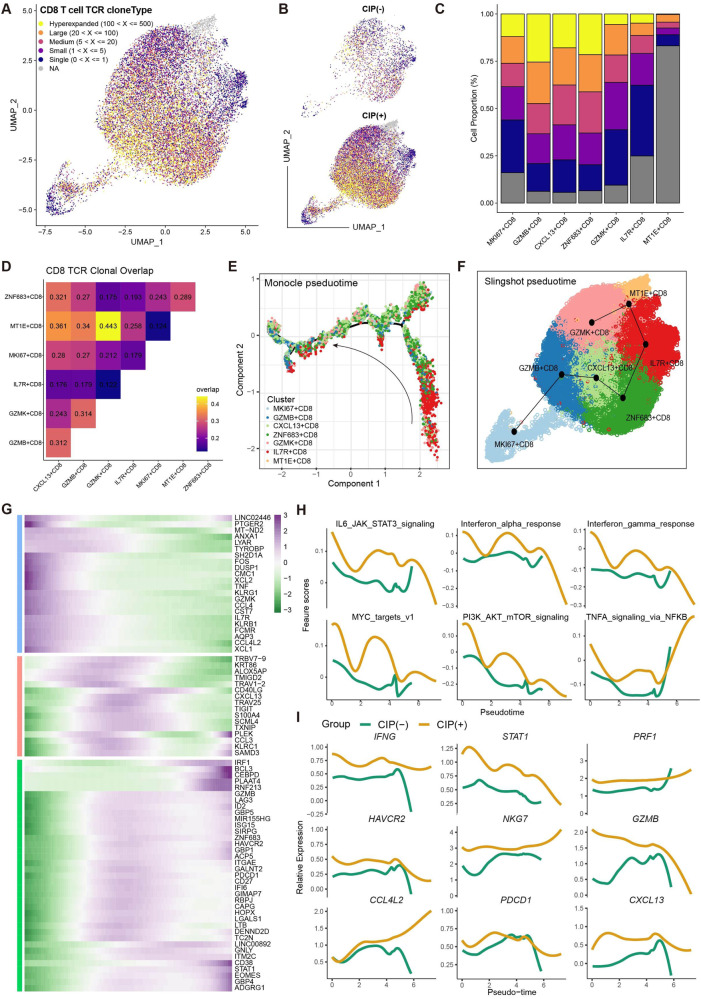
TCR and trajectory analysis of CD8 T cell subclusters in CIP+ and CIP− samples. UMAP plot showing TCRαβ clonality for each CD8T cell subset (**A**), and pathology (**B**) estimated by scRepertoire. **C** Bar plot showing the percentage of TCR clonality types distribution across each CD8T cell subset. **D** Heatmap showing the TCR clonality types overlap among each CD8T cell subset. **E** monocle showing the differentiation trajectory among each CD8T cell subset. **F** Sling shot showing the differentiation trajectory among each CD8T cell subset. **G** Heatmap showing dynamic changes in gene expression along the pseudotime of CD8T cell differentiation trajectory. **H** Two-dimensional plots showing the dynamic expression of Hallmark geneset scores along with the pseudotime in CIP+ and CIP− groups. The values of the y axis are the calculated GSVA scores. **I** Two-dimensional plots showing the dynamic expression of significantly upregulated genes along with the pseudotime in CIP+ and CIP− groups.

Analysis of transcriptomes for 30 762T/NK cells generated 12 subclusters (Supplementary Fig. 2A–C), and the cytotoxic effectors GZMK+ CD8, GZMB+ CD8, CXCL13+ CD8, and proliferative CD8 showed a dramatic increase in the CIP+ group compared to the CIP− group (Supplementary Fig. 2C, D). Next, we performed a further detailed clustering of CD8+ T cells and generated seven subclusters of CD8+ T cell subtypes: ZNF683+ CD8, GZMB+ CD8, GZMK+ CD8, IL7R+ CD8, CXCL13+ CD8, MT1E+ CD8, and proliferating MKI67+ CD8 (Fig. 2A, B, Supplementary Table S7). The organization of the CD8+ T cell compartment showed great differences between CIP− and CIP+ group (Fig. 2C); GZMB+ CD8, CXCL13+ CD8, and proliferating MKI67+ CD8 were more frequent in CIP+ samples, whereas naïve IL7R+ CD8 and resident ZNF683+ CD8 were more enriched in CIP− samples (Fig. 2D). Flow cytometry analysis confirmed that CIP associated CD8+ T cell subclusters increased at the protein level (Fig. 2E). Interestingly, the immune-checkpoint (LAG3, HAVCR2, PDCD1), immune cell-homing signals (CXCL13, CCL3/4), IFNγ response (IFNG) and cytotoxic effector (GZMB, PRF1, GNLY) markers were the top differentially expressed genes (DEGs) in CIP associated CD8+ T cell subclusters (Fig. 2F, G). Gene set enrichment analysis (GSEA) of the upregulated genes showed that the differentially upregulated genes in the CIP associated CD8+ T cell subclusters compared to other CD8+ T cell subclusters were enriched in interferon signaling, IFNG, and lung proliferating NK/T cell pathways, which correlated with T cell effector function (Fig. 2H), indicating a highly cytotoxic and effective environment for CIP induced by ICIs. To investigate the origin of the CXCL13+ CD8+ T cell subcluster, we performed a trajectory analysis and a T-cell receptor (TCR) clone expansion analysis. The corresponding TCR sequences revealed that GZMB+ CD8, CXCL13+ CD8 shared expanded TCR clonotypes with resident ZNF683+ CD8, with the highest clonality observed in CXCL13+ CD8 cells (Fig. 3A-D, and Supplementary Fig. 2E), In addition, CIP+ group exhibited more hyperexpanded TCR clonotypes than CIP− group, although the trend is not notably (Fig. 3B, and Supplementary Fig. 2F), indicated that CD8+ T cells undergo clonal expansion following TCR activation in patients with CIP. Furthermore, the monocle (Fig. 3E) and slingshot (Fig. 3F) pseudotime trajectory analysis showed a possible differentiation trajectory from naïve IL7R+ CD8 into resident ZNF683+ CD8 and then into cytotoxic CD8+ T cell subsets: CXCL13+ CD8 and GZMB+ CD8 (Fig. 3F, G), which is consistent with the differentiation trajectory in which ZNF683+ CD8 contributes to cytotoxic CD8+ T cells in colitis induced by ICI therapy [30]. Pathway analyses revealed upregulation of interferon (IFN) responses, inflammatory responses, MYC, PI3K-AKT-mTOR, and TNF pathways along the CD8+ T cell differentiation trajectory in CIP+ samples compared to CIP− samples (Fig. 3H). Interestingly, the expression of immune-checkpoint (HAVCR2, PDCD1), immune cell-homing signals (CXCL13), IFNγ response (IFNG, STAT1) and cytotoxic effector (GZMB, PRF1, NKG7) markers increased alongside the trajectory (Fig. 3I). Therefore, our results highlighted the dynamic gene expression and pathway patterns along trajectories, and clonal expansion of CD8+ T cell subclusters associated with CIP.

### Characteristics and dynamics of CD4+ T cells in BALF with CIP

**Fig. 4:**
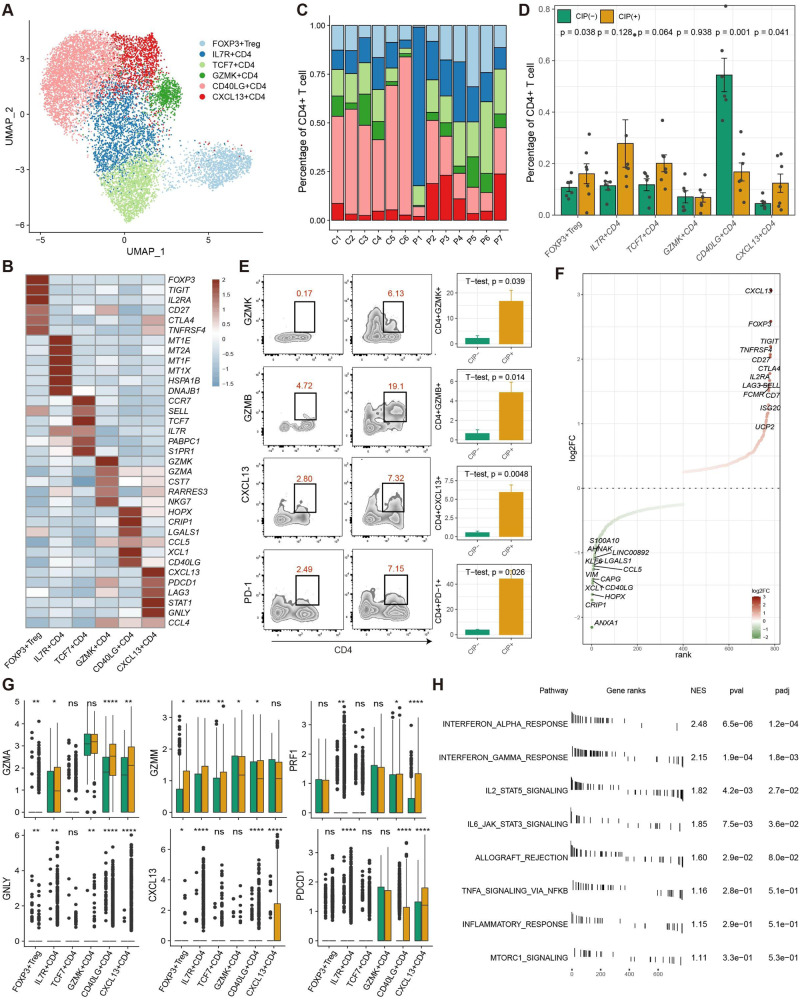
CIP associated CD4+ T Cell with cytotoxic effector programs. **A** Sub-clustering of CD4+ T cells revealed 6 cell-subclusters. **B** Heat map showing the scaled expression of top 6 differentially expressed genes among six CD4+ cell-subclusters. **C** The proportion of sample contributions per annotated CD4+ cell-subclusters. **D** Relative contribution of six CD4+ cell-subclusters in distinct sample pathology. *P* values were assessed by Student’s *t* test. **E** CIP associated CD4+ T frequencies as determined by flow cytometry (percentage of live CD4+ cells), *P* values were assessed by Student’s *t* test. **F** Ranking of significantly differentially expressed genes in CIP associated CD4+ T cell clusters compared with other CD4+ T cell clusters. **G** Box plots showing the expression of immune-checkpoint (PDCD1), immune cell-homing signals (CXCL3), effector (GNLY) and cytotoxic (GZMA, GZMM, PRF1) markers in CD4+ cell-subclusters in distinct pathology samples. *P* values were assessed by Wilcoxon test, *P < 0.05, **P < 0.01, ***P < 0.001. **H** Functional enrichment analysis showing significant hallmark gene sets enriched in CIP associated CD4+ T cell clusters compared with other CD4+ T cell clusters.

**Fig. 5:**
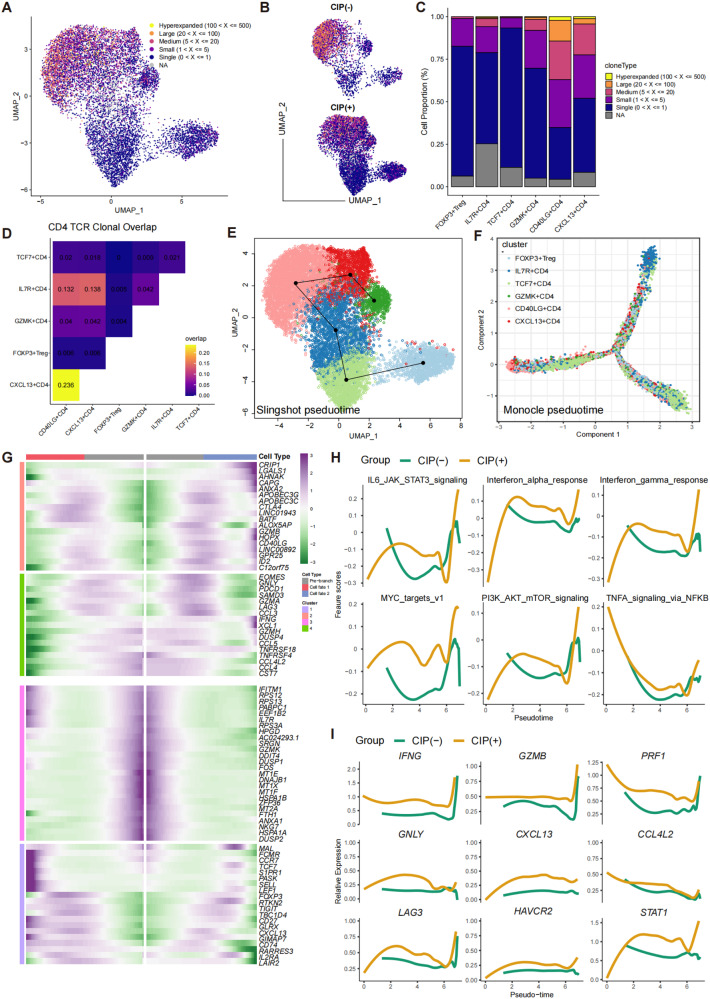
TCR and trajectory analysis of CD4 T cell subclusters in CIP+ and CIP− samples. UMAP plot showing TCRαβ clonality for each CD4T cell subset (**A**), and pathology (**B**) estimated by scRepertoire. **C** Bar plot showing the percentage of TCR clonality types distribution across each CD4T cell subset. **D** Heatmap showing the TCR clonality types overlap among each CD4T cell subset. **E** Slingshot showing the differentiation trajectory among each CD4T cell subset. **F** monocle showing the differentiation trajectory among each CD4T cell subset. **G** Heatmap showing dynamic changes in gene expression along the pseudotime of CD4T cell differentiation trajectory. **H** Two-dimensional plots showing the dynamic expression of Hallmark geneset scores along with the pseudotime in CIP+ and CIP− groups. The values of the y axis are the calculated GSVA scores. **I** Two-dimensional plots showing the dynamic expression of significantly upregulated genes along with the pseudotime in CIP+ and CIP− groups.

Sub-clustering of total CD4+ T cells generated six subclusters of CD4+ T cells (Fig. 4A), annotated as TCF7+ CD4, GZMK+ CD4, CD40LG+ CD4, IL7R+ CD4, CXCL13+ CD4, and FOXP3+ Treg, according to the top five DEGs (Fig. 4B, Supplementary Table S8). Consistent with the results for CD8+ Tcell subclusters, the proportion of CD4+ T cell subclusters differed dramatically among samples (Fig. 4C). CD40LG+ CD4+ T cell subcluster was predominant in the CIP− group, whereas TCF7+ CD4, FOXP3+ Treg, and CXCL13+ CD4 were more frequent in the CIP+ group (Fig. 4D), and the percentage of CIP associated CD4+ T cell subclusters was validated by flow cytometry analysis at the protein level, indicated that GZMB, GZMK, CXCL13, PDCD1(PD1) was highly expressed in CIP+ samples (Fig. 4E). Furthermore, the DEGs analysis revealed that the immune-checkpoint genes (CTLA4, TIGIT, LAG3), immune cell-homing signals genes (CXCL13) were both upregulated in CIP associated CD4+ T cells subclusters (Fig. 4F). Also, the cytotoxic effector marker (GZMA, GZMB, PRF1, GNLY) expressed a high level in CD4+ T cells subclusters of CIP+ group than CIP− group (Fig. 4G). We also observed that CIP associated CD4+ T cells were enriched in the inflammation signaling pathways such as IFN, TNF, IL2, and IL6 (Fig. 4H), indicated a hyper inflammatory status in CIP samples. Furthermore, the TCR sequences showed that CXCL13+ CD4 and CD40LG+ CD4 had more hyperexpanded TCR clonotypes (Fig. 5A–C) and CXCL13+ CD4 shared more expanded TCR clonotypes with CD40LG+ CD4 and IL7R+ CD4 (Fig. 5D), indicating that IL7R+ CD4 contributed to differentiate to CD40LG+ CD4 to CXCL13+ CD4 in CIP+ samples, whereas it differentiated to CD40LG+ CD4 but not CXCL13+ CD4 in CIP− samples. Further pseudotime trajectory analysis confirmed this hypothesis (Fig. 5E, F): naïve IL7R+ CD4 cells have two differentiation routines, contributing to CXCL13+ CD4, CD40 LG+ CD4, and Treg cells (Fig. 5E–G), indicating the phenotypic transition of naïve CD4+ T cells to effector status may be an important phenomenon in CIP+ samples. Pathway analyses revealed upregulation of IFN responses, inflammatory responses, PI3K-AKT-mTOR, MYC, and TNF pathways along the CD4+ T cell differentiation trajectory in CIP+ samples compared to CIP− samples (Fig. 5H). Furthermore, the expression of immune-checkpoint (LAG3), immune cell-homing signals (CXCL13, CCL4L2), IFNγ response (IFNG), and cytotoxic effector (GZMB, PRF1, GNLY) markers increased alongside the cellular differentiation trajectory in CIP+ samples (Fig. 5I). Therefore, our results highlighted the gene and pathway expression patterns along trajectories, and clonal expansion of CD4+ T cell subclusters associated with CIP.

### Hyperinflammatory phenotype of myeloid cells expanded in BALF with CIP

**Fig. 6:**
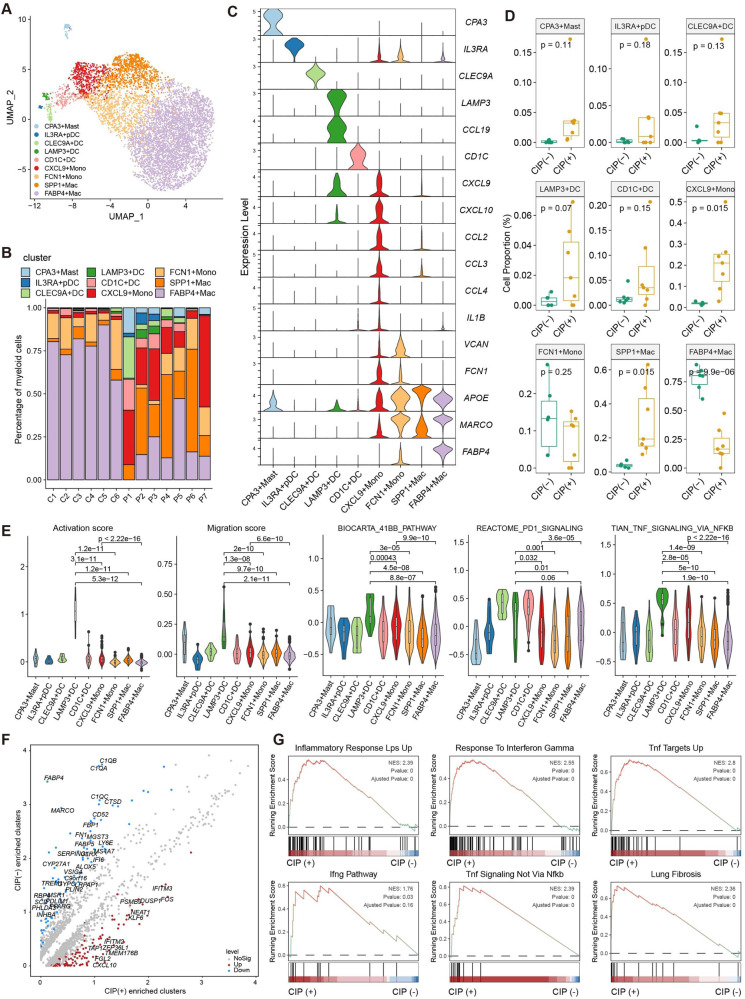
Hyperinflammatory myeloid cells expanded in CIP. **A** Sub-clustering of myeloid cells revealed nine cell-subclusters. **B** The proportion of sample contributions per annotated myeloid cell-subcluster. **C** Expression of representative cell type signature among nine myeloid cell-subclusters. **D**, Relative contribution of nine myeloid cell cell-subclusters in distinct sample pathology. *P* values were assessed by Student’s *t* test. *P < 0.05, **P < 0.01, ***P < 0.001. **E** Expression of migration, maturation, immune checkpoints, and TNF pathway score in nine myeloid cell cell-subclusters, *P* values were assessed by Student’s *t* test. *P < 0.05, **P < 0.01, ***P < 0.001. **F** Volcano map showing the upregulated genes between CIP+ associated myeloid cells subclusters and CIP− associated myeloid cells subclusters. **G** GSEA plots showing pathways enriched in CIP associated myeloid cell subclusters compared with other myeloid cell subclusters.

**Fig. 7:**
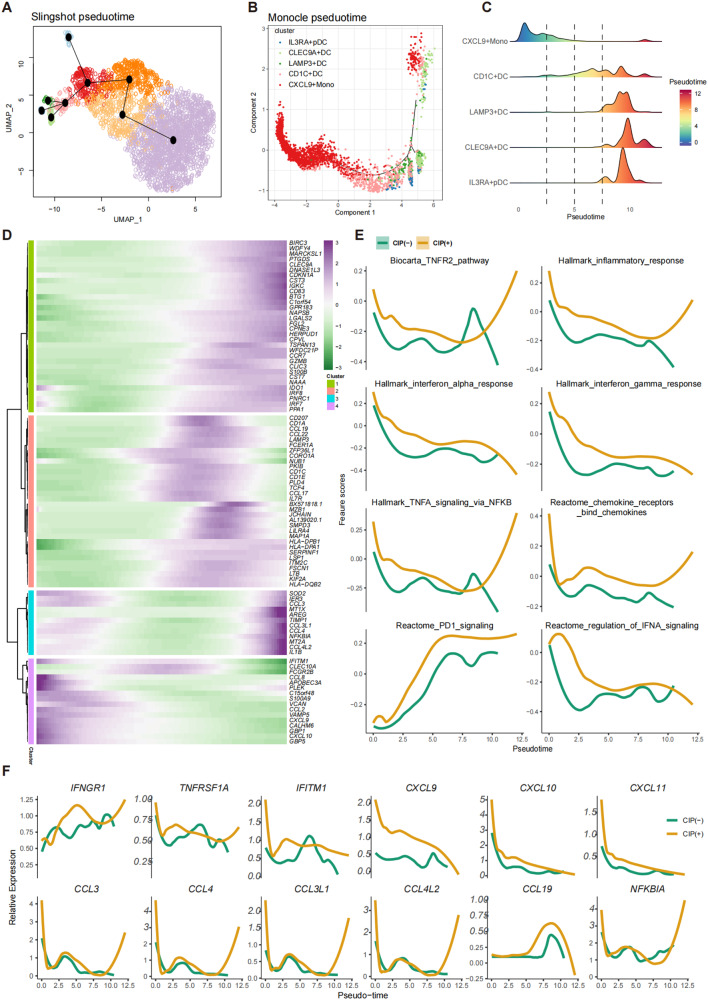
Trajectory analysis of myeloid cell subclusters in CIP+ and CIP− samples. **A** Slingshot showing the differentiation trajectory among each myeloid cell subset. **B**, **C** Monocle2 showing the differentiation trajectory among monocytes and DC subsets. **D** Heatmap showing dynamic changes in gene expression along the pseudotime of monocytes and DC subsets differentiation trajectory. **E** Two-dimensional plots showing the dynamic expression of Hallmark geneset scores along with the pseudotime in CIP+ and CIP− groups. The values of the y axis are the calculated GSVA scores using Hallmark geneset. **F** Two-dimensional plots showing the dynamic expression of significantly upregulated genes along with the pseudotime in CIP+ and CIP− groups.

Myeloid cell related molecular characteristics and differentiation trajectory abnormalities have been associated with COVID-19 and other diseases [33]. In this study, myeloid cells accounted for over 50% of the total sequenced cells, which were subclassified into eight cell subtypes and annotated as CPA3+ Mast cell, CLEC9A+ (DC), CD1C+ DC, LAMP3+ DC, IL3RA+ DC, CXCL9+ monocyte, FCN1+monocyte, SPP1+ and FABP4+macrophage (Fig. 6A–C, Supplementary Fig. 3A, Supplemental Table S9). The proportion of these cell subtypes differed notably among patients (Fig. 6B), and CXCL9+ and SPP1+ monocyte was more frequent in the CIP+ samples than in the CIP− samples, whereas the number of FABP4+ macrophages decreased dramatically in the CIP+ samples (Fig. 6D, *p* < 0.05). CXCL9+ monocytes express the proinflammatory-chemokines IL1B, CCL2/3/4, and CXCL9/10, indicating a hyperinflammatory phenotype. The LAMP3+ DCs also showed higher percentage in the CIP+ samples although the *p* value was not statistically significant (*p* = 0.07), which expressed the DC maturation markers such as LAMP3 and CD83, the lymphocyte recirculation chemokine CCL19, and the migration marker CCR7 (Fig. 6C, Supplementary Fig. 3A, Supplementary Table S9). GSEA revealed LAMP3+ DCs exhibited high maturation and migration scores (Fig. 6E, Supplementary Table S10), indicating the potential of migration from tissue to BALF and providing information on the mechanisms of CIP pathogenesis. LAMP3+ DCs also enriched immune-checkpoint signaling pathways such as 41BB (CD137), PD1, CTLA4, and effector T cell function-associated pathways such as TNF and IFNG (Fig. 6E, Supplementary Fig. 3A, B). Furthermore, the myeloid cell subclusters of CIP+ samples showed higher expression of IFN pathway genes, such as CXCL9, CXCL10, IFITM2, and IFIT3, than the myeloid cell subclusters of CIP− samples (Fig. 6F). GSEA showed that DEGs in the CIP associated myeloid cell subclusters were enriched in inflammation pathway such as IFN, IL2, IL6, TNF signaling, and lung fibrosis (Fig. 6G, Supplementary Fig. 3B). These results were validated by the chemokine assay (Supplementary Fig. 3D, E). The pseduotime trajectory analysis revealed that the differentiation of hyperinflammatory CXCL9+ monocytes to anti-inflammatory alveolar macrophages was blocked, whereas LAMP3+ DCs with high migration characteristics was highly expanded in CIP, which was derived from the differentiation of CXCL9+ monocytes (Fig. 7A–D, Supplementary Fig. 4A–C). Furthermore, pathway analyses revealed the upregulation of IFN responses, inflammatory response, and TNFR2, TNFA, and PD1 pathways along the myeloid differentiation trajectory in CIP+ samples (Fig. 7E). Gene expression profiling along the trajectories also identified IFN signaling associated genes such as CXCL9/10/11, IFITM1, IFNGR1, and TNFRSF1A as important activated markers (Fig. 7F) to distinguish CIP+ from CIP− samples.

### Multilineage intercellular crosstalk and potential therapeutic targets for CIP

**Fig. 8:**
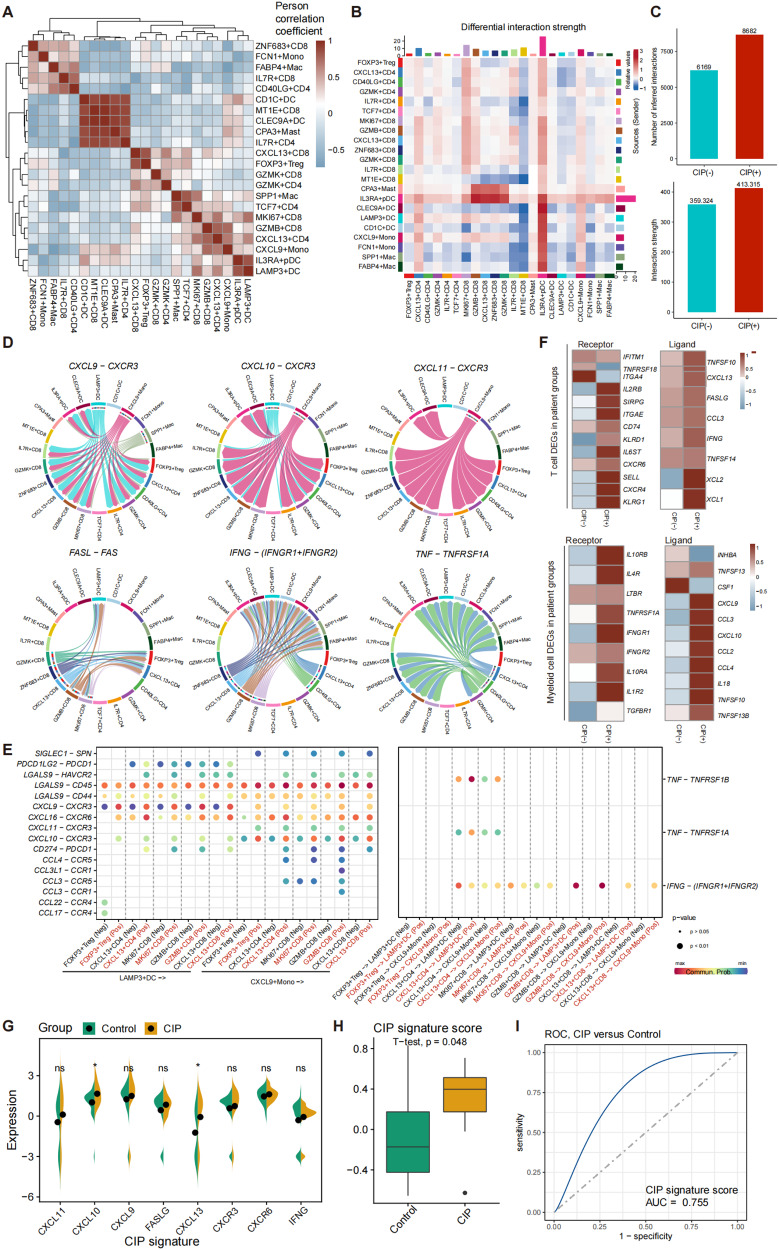
Multi-lineage intercellular crosstalk and potential therapeutic targets for CIP. **A** Heatmap showing the Person correlation coefficient of cell types abundance. **B** Differential number of interactions in different BALF immune cell subtypes. **C** Number of inferred interactions and scores in distinct BALF sample pathology. **D** Circle plots showing the significant ligand-receptor pairs between CXCL13+ T cells, CXCL9+ monocytes and LAMP3-DC. **E** Dot plot showing the communication probability of the indicated ligand-receptor pairs between CXCL13+ T cells, CXCL9+ monocytes and LAMP3-DC. **F** Heatmap showing averaged expression of the indicated cytokine and cytokine receptor genes compared among patient groups. **G**, **H** Violin plot showing the expression of CIP associated genes (**G**), and signature (**H**) in CIP+ lung tissue and CIP− lung tissue. **I** the diagnostic power of CIP associated signature for classification of CIP+ lung tissue and CIP− lung tissue. *P* values were assessed by the Student’s *t* test.

Having defined CIP associated T cell and myeloid cell subpopulations, obvious correlations between the abundance of CXCL9+monocytes, LAMP3+ DCs and CIP associated T cell subclusters were found, such as CXCL13+ CD4, CXCL13+ CD8, and GZMB+ CD8 (Fig. 8A, Supplementary Fig. 5). We performed an unbiased ligand-receptor interaction analysis between these populations to explore the multilineage intercellular crosstalk associated with CIP pathogenesis. Numerous ligand-receptor pairs and signaling pathway networks were detected (Fig. 8B, Supplementary Fig 6A). The CIP+ group exhibited more ligand-receptor pairs than the CIP− group (Fig. 8C), and CXCL9+ monocytes and LAMP3+ DCs showed close interaction links with CXCL13+ T cells (Fig. 8B, D). Specifically, the immune checkpoint inhibitor PD-L1, PD-L2, and TIGIT signaling pathways and the CD80 signaling pathway networks were enriched between CXCL9+ monocytes, LAMP3+DCs and CXCL13+ T cells (Supplementary Fig. 6B). Furthermore, CXCL9+ monocytes and LAMP3+ DCs interacted closely with CXCL13+ T and GZMB+ T cells through CXCL9/10/11-CXCR3, FASLG-FAS, IFNG-IFNGR1/IFNGR2, and TNF-TNFRSF1A ligand-receptor pairs (Fig. 8D). Previous studies have reported that PD-L1 expression in macrophages and DCs may be closely related to ICIs efficacy [34]. In this study, the CIP+ samples exhibited a stronger interaction between PD-L1, PD-L2, and TIGIT signaling than the CIP− samples (Fig. 8E), indicating that the CIP+ group may benefit more from ICIs therapy. In addition, the CIP associated T cells expressed high levels of the chemokine receptor genes CXCR4 and CXCR6 (Fig. 8F), with the myeloid cell ligands CXCL9/10/11 expressed in CIP associated monocytes and DCs (Fig. 8D, E). The T cell subclusters exhibited elevated expression levels of effector and cytotoxic genes such as IFNG, GZMB, GNLY, and PRF1 in CIP+ samples (Figs. 2G, 4G, and 8F), and CXCL9+ monocytes and LAMP3+ DCs of CIP+ samples expressed high levels of the receptor genes IFNGR1/2 and TNFRSF1A/B (Fig. 8E, F). Therefore, we hypothesized that CXCL9+ monocytes and LAMP3+ DCs contributed to CIP pathogenesis by recruiting effector and cytotoxic T cell through the CXCL9/10/11-CXCR3 pathway. Furthermore, a panel of T cell and myeloid cell featured molecules, such as CXCL10, CXCL13 were also elevated in CIP+ lung tissues [27] than CIP− lung tissues (Fig. 8G), indicating the potential of BALF makers for screening CIP. We further developed a CIP signature (CXCL9/10/11/13, CXCR3/6, FASLG, and IFNG), and found that the CIP signature expressed a high level in CIP+ tissue samples than CIP− tissue samples (Fig. 8H), and achieved a high diagnostic power to distinguish CIP+ tissue sample from CIP− tissue samples (Fig. 8I). Therefore, our unbiased dissection of key ligand-receptor interactions between CIP associated hyperinflammatory myeloid cell subclusters and effector T cell subclusters highlights CXCL, IFN, FAS, and the TNF signaling axis as important regulators within the BALF microenvironment of patients with CIP, which is also an important biomarker for the screening of CIP.

## Discussion

IrAEs caused by ICIs can complicate effective therapy and limit these use in patients with cancer [35, 36]. Understanding the precise pathophysiology associated with irAEs is critical for developing noninvasive developing diagnostic and therapeutic strategies for CIP. In this study, we used BALF, a noninvasive reproducible sampling method, to generate a comprehensive and systematic single-cell transcriptomic atlas of CIP. We identified the key cellular players, molecular pathways, and effector programs associated with CIP. Furthermore, the trajectories for both T cells and myeloid cells, as well as TCRαβ analysis, demonstrated the cellular differentiation programs and clonality expansion associated with CIP. We are the first to use scRNA-seq and scTCR-seq to present a transcriptomic atlas of the bronchoalveolar immune landscape in CIP, comparing immune cell types as well as their precise phenotype and clonotype distribution to those of ICIs treated patients without CIP, and validated these results with flow cytometry and cytokine expression analysis. Recently, a study conducted by Franken et al. [37] have performed deep immune profiling using scRNA-seq and scTCR-seq to discover the mechanism of CIP; however, they compared CIP patients with ICI-untreated cancer patients, which may not reflect the real differences in cellular and molecular characteristics of irAEs induced by ICIs therapy.

Here, we demonstrated a dramatic accumulation of CXCL13+ CD4+ T and CXCL13+ CD8+ T cells, characterized by high expression levels of the cytotoxic effector signatures IFNG, GZMB, and PRF1, which supports previous findings that CD4+ and CD8+ T cell accumulation are hallmarks of CIP [25, 27, 37]. Recent studies have shown that elevated frequencies and activation of CXCL13+ T cells associated with an effective response to ICIs therapy [38–40]. TCR clonal expansion analysis revealed that CXCL13+ T cells exhibited hyperexpanded TCR clonotypes, and pseudotime analysis revealed a potential differentiation trajectory from naïve to cytotoxic effector status, highlighting the important role of cellular phenotypical transition alongside CIP initiation and progression. Therefore, we hypothesized that the activation of naïve T cells played an important role in CIP and that the activation of such T cells induced the subsequent recruitment of additional CD8+ and CD4+ T cell populations from the blood.

Mast cells are tissue-resident, innate immune cells that play a key role in the inflammatory response [41]. Activation of mast cells by immunotherapy can induce pneumonitis [42]. In this study, we observed an enrichment of mast cells in the CIP+ group. Meantime, CXCL9+monocytes and LAMP3+DCs were also elevated in CIP, and pseduotime trajectories showed that LAMP3+DCs were derived from the differentiation of hyperinflammatory CXCL9+monocytes, whereas the differentiation routine of anti-inflammatory alveolar macrophages was blocked, indicating a hyperinflammatory environment in CIP samples [25, 37]. Importantly, LAMP3+DCs expressed the lymphocyte recirculation chemokine CCL19, the maturation markers LAMP3 and CD83, and the migration marker CCR7. GSEA revealed that LAMP3+DCs exhibited high maturation and migration scores, suggesting that LAMP3+DCs have the potential to migrate from tumors to BALF, which supports previous findings that LAMP3+DCs can migrate from tumors to hepatic lymph nodes [43]. With our results, we speculate that LAMP3+DCs may migrate from the lung tumor site to the BALF, which could be a potential liquid biomarker to monitor CIP.

The abundance of CXCL9+ monocytes and LAMP3+ DCs was also notably correlated with cytotoxic effector CXCL13+ CD4+ T, CXCL13+ CD8+ T, and GZMB+ CD8+ T cells. Multilineage intercellular crosstalk analysis revealed that CXCL9+monocytes and LAMP3+DCs interacted closely with the cytotoxic effectors CXCL13+ T and GZMB+ CD8+ T cells through CXCL9/10/11-CXCR3, FASLG-FAS, IFNG-IFNGR1/IFNGR2, and TNF-TNFRSF1A ligand-receptor pairs. Interestingly, IFN-α or IFN-γ response and TNF-α genes (IFNG, STAT1, TNF) were highly expressed in CIP associated CD4+ and CD8+ T cell clusters alongside pseudotime of the T cell differentiation trajectory, with the receptor IFNGR1/2, TNFRSF1A increased alongside pseudotime of the myeloid cell differentiation trajectory. IFN-γ can directly induce epithelial cells apoptosis and lead to inflammatory bowel disease pathogenesis [44]. TNF is a crucial proinflammatory cytokine in a wide range of inflammatory diseases [45]. CXCL9/10, the ligands for CXCR3 and induced by IFNγ, were overexpressed by monocytes in patients with CIP versus patients without CIP, which was also enriched in patients with colitis induced by ICIs [30], are important for recruiting effector T cells to tumors, which would be expected to have positive effects on anti-tumor immunity [46]. The FASLG-FAS pathway is a representative system of apoptosis signaling ligand-receptor molecules that may play an important role in the pathogenesis of fibrosing lung diseases [47]. Therefore, CXCL9+monocytes and LAMP3+DCs may be key regulators and cellular players that led to the recruitment of cytotoxic effector T cells by upregulating of IFN-α, IFN-γ, TNF-α, CXCL and FAS signaling. These results indicated that the blockade of IFN-α, IFN-γ, TNF-α, CXCL and FAS signaling might be effective in modulating the inflammatory response induced irAEs by ICIs.

This study had several shortcomings, including a limited number of heterogeneous patients and a lack of longitudinal samples collected before and after the occurrence of CIP. Secondly, our BALF results need to be validated in a large independent patient cohort using tissue scRNA+TCR-seq. However, this single-cell transcriptome and the TCR landscape of bronchoalveolar immune cells reveal an in-depth understanding of the mechanisms underlying immunopathogenesis in CIP and further support the possibility of using immune cells in BALF as biomarkers in the prediction of CIP.

## Methods

### Study population

Patients diagnosed with NSCLC and treated with ICIs were enrolled in this prospective observational study. BALF was collected whenever patients underwent a bronchoscopy. Samples were obtained before the initiation of steroids and antibiotics. A clinical diagnosis of CIP was adjudicated by the multidisciplinary irTox team. If CIP was diagnosed, the BALF sampled was categorized as “CIP”. After adjudication, the patients with CIP were treated with high-dose steroids (1 mg/kg prednisone). Second-line agents (tocilizumab) were added at the discretion of the treatment team if no improvement was observed after 72 h.

### CIP diagnosis

CIP was defined as shortness of breath, decreased exercise tolerance, exertional desaturation, and/or cough, along with the presence of new radiographic infiltrates and a lack of evidence of infection (negative cultures on BALF, negative respiratory viral swab) or alternate etiologies (diffuse alveolar hemorrhage, heart failure) [25]. Radiographic assessment was performed based on the Response Evaluation Criteria in Solid Tumors (RECIST); cases where new infiltrates were considered to represent tumor progression or radiation pneumonitis were excluded from both the control and CIP groups. The diagnosis of CIP was adjudicated following a review and discussion of the pertinent microbiologic and radiographic [17, 48] data by two oncologists and a radiologist. Patients in whom clinical requirements regarding infection were present (e.g., clinical presence of fever, purulent sputum, sick contacts, and elevated bands on complete blood count differential) were not adjudicated for CIP, even if the BALF cultures were negative. CIP was graded based on the National Cancer Institute’s Common Terminology Criteria for Adverse Events (CTCAE), version 4.03.10. The radiological patterns of CIP was described according to the study by Naidoo et al. [49].

### BALF collection

In the control patients, the unaffected middle lobe (without tumor) was lavaged. In patients with CIP, areas with new infiltrates that were not associated with the tumor were lavaged. Volumes of instilled and returned saline were extracted from the BALF procedure notes. The BALF specimens were processed using an ammonium chloride–potassium lysis solution.

### Isolation of BALF cells

Approximately 20 ml of BALF was collected and placed on ice. The BALF was processed in 2 h. After passage of BALF through a 100-μm nylon cell strainer to remove clumps and debris, the supernatant was centrifuged, and the cells were re-suspended in cooled RPMI 1640 complete medium. The supernatant samples were collected and stored at −80 °C until use.

### Single-cell sequencing and pre-processing data

We prepared the single-cell RNA-seq and TCR libraries on the Chromium platform (10× Genomics), using the Chromium Next GEM Single-Cell 5’ Kit v2. The FACS sorting live cells (7-AAD negative) were pooled together and washed with RPMI-1640 three times, concentrated to 600–1 000 cells per μL, then immediately loaded on a 10× microfluidic chip (10x Genomics) following the manufacturer’s protocol to generate a complementary deoxyribonucleic acid (cDNA) library in Capitalbio Technology Corporation (China). Amplified cDNA was then used for both 50 gene expression library construction, and TCR V(D)J targeted enrichment was performed using the Chromium Single-Cell V(D)J Enrichment Kit, Human T cell (10x Genomics), followed by V(D)J library construction. Raw sequencing data were aligned to the GRH38 reference genome using the Cell Ranger (10X Genomics, v4) count and vdj functions. The count matrices of gene expression from each sample were imported into Seurat [50]. We selected high-quality cells for further analysis following three criteria:1) cells had over 2 001 unique molecular identifiers (UMIs), fewer than 6 000, over 301 expressed genes or fewer than 10% UMIs derived from the mitochondrial genome. 2) Genes were expressed in over 10 cells in a sample. 3) Cell doublets were removed using the DoubletFinder [51] R package. The cell-by-gene expression matrices of the remaining high-quality cells were integrated using the RunFastMNN function provided by the SeuratWrappers R package and normalized to the total cellular UMI count. The union of the top 2 000 genes with the highest dispersion in each dataset was used to generate an integrated matrix. We then performed data normalization, dimension reduction, and cluster detection, as previously reported. Briefly, gene expression matrices were scaled by regressing the total cellular UMI counts and the percentage of mitochondrial genes. Principal component analysis (PCA) was conducted using highly variable genes (HVGs) and the top 30 significant principal components (PCs) were selected for the Uniform Manifold Approximation and Projection (UMAP) dimension reduction, and visualization of gene expression. We annotated cell subclusters with gene expression patterns similar to those of the same cell type, and the cell types in the resulting two-dimensional representation were annotated to known biological cell types using canonical marker genes.

### Trajectory analysis

To explore potential differentiation routines among CD4+ T cells, CD8+ T cells, and myeloid cell subtypes, we performed trajectory analysis via the monocle [52] and Slingshot [53] packages. For monocle trajectory inference analysis, we constructed the monocle object using “newCellDataSet” function, and the DEGs calculated using the “differentialGeneTest” function were selected for the trajectory analysis. Then, the “DDRTree” function was used for dimensionality reduction and the “plot_cell_trajectory” function for visualization. For Slingshot [53] trajectory inference analysis, Slingshot was used and PCA-based dimension reduction was performed with DEGs, followed by two-dimensional visualization with UMAP.

### Pathway and signature enrichment analysis

To illustrate the enriched signaling pathways of T cells and myeloid subtypes, we used the GSVA [54] package to assess pathway differences using the hallmark gene set and the C2 gene set provided by the Molecular Signatures Database (MSigDB), which were calculated with a linear model offered by the limma package. We used the GSEA package to calculate the distribution of gene sets in lists of genes ordered by population expression differences. We also employed GSVA to evaluate the expression levels of CIP-associated signatures (CXCL9/10/11/13, CXCR3/6, FASLG, and IFNG) in 8 CIP+ lung tissues and 29 normal controls [27].

### Intercellular crosstalk

We used the Cellchat [55] package (v0.0.2) to infer intercellular communication and significant ligand-receptor pairs that participate in CIP immunopathogenesis, following a standard pipeline implemented in R (https://github.com/sqjin/CellChat). We set the ligand-receptor interaction list for human and projected the gene expression data onto a protein-protein interaction (PPI) network by identifying the overexpressed ligand-receptor interactions. To obtain biologically significant cell-cell communication, the probability values for each interaction were calculated by performing permutation tests. The inferred intercellular communication network of each ligand-receptor pair and each signaling pathway was summarized and visualized using circle plots and heatmaps.

### TCR analysis

We used the scRepertoire [56] R package to analyze the TCR clonality of CD4+ T cell and CD8+ T cell, briefly, the filtered_contig_annotations output from Cell Ranger was imported in Seurat to create a list object of TCR genes and CDR3 sequences by cell barcodes using combineTCR function. The combineExpression function was used to integrate clonotype information with the filtered Seurat object. The frequency of the clonotypes was binned by the patient using the following parameters: single = 1, small = 5, medium = 20, large = 100, hyperexpanded = 500. The clonotypes were determined using VDJC genes comprising the TCR. The distribution of the clonotype bins was visualized using the Seurat DimPlot function.

### Definition of the phenotypes of myeloid cell subclusters

Phenotypic scores were defined as the average expression levels of the signature genes. The signature genes [43] used to define the M1/M2 phenotype scores of the macrophage clusters and the migration and activation scores of the DC clusters are listed in the Supplementary Table 10.

### Cytokine measurement by Luminex and statistic

Cytokines that include 6Ckine/CCL21, BCA-1/CXCL13, CTACK/CCL27, ENA-78/CXCL5, Eotaxin/CCL11, Eotaxin-2/CCL24, Eotaxin-3/CCL26, Fractalkine/CX3CL1, GCP-2/CXCL6, GM-CSF, Gro-α/CXCL1, Gro-β/CXCL2, I-309/CCL1, IFN-ϒ, IL-1β, IL-2, IL-4, IL-6, IL-8 / CXCL8, IL-10, IL-16, IP-10/CXCL10, I-TAC/CXCL11, MCP-1/CCL2, MCP-2/CCL8, MCP-3/CCL7, MCP-4/CCL13, MDC/CCL22, MIF, MIG/CXCL9, MIP-1α/CCL3, MIP-1δ/CCL15, MIP-3α/CCL20, MIP-3β/CCL19, MPIF-1/CCL23, SCYB16/CXCL16, SDF-1α+β/CXCL12, TARC/CCL17, TECK/CCL25, TNF-α were detected in CIP+ and CIP− BALF samples according to the instruction (Bio-Rad, Bio-Plex Pro™ Human Chemokine Panel, 40-Plex), each group contains over five samples. The expression matrix of cytokines is listed in Supplementary Table 11 and was scaled and clustered using complete linkage clustering and Euclidean distance using a heatmap. A Student’s *T* test was used to compare the mean differences between the control and CIP cytokine values. Statistical significance was established at *p* < 0.05.

### Flow cytometry

To validate the scRNA-seq findings, the following antibodies were used: anti-human CD45 (AmCyan, 339203, Biosciences), Anti-Human CD3 (Pacific Blue, 300330, Biolegend), anti-human CD4 (PE/Cyanine7, 300512, Biolegend), anti-human CD8a (APC/Cyanine7, 300926, Biolegend), Anti-Human CD274 (APC, 329707, Biolegend), Anti-Human CXCL13 (APC, WA3170553, Invitrogen), anti-human/mouse Granzyme B (FITC, 515403, Biolegend), anti-human Granzyme K (FITC, 370507, Biolegend), Anti-Ki-67 (PE, 556027, Biosciences), anti-human CD68 (PE, 333808, Biolegend), κ isotype Ctrl (PE, 400112, Biolegend), κ isotype Ctrl (FC) (APC, 400122, Biolegend), κ isotype Ctrl (FITC, 981802, Biolegend). Stained cells were detected using a BD FACSCanto^TM^ II and analyzed using FlowJo software.

### Supplementary information

supplementary figures and tables legends

Supplementary figures

Supplementary Table 1

Supplementary Table 2

Supplementary Table 3

Supplementary Table 4

Supplementary Table 5

Supplementary Table 6

Supplementary Table 7

Supplementary Table 8

Supplementary Table 9

Supplementary Table 10

Supplementary Table 11

## Data Availability

The raw scRNA-seq data of the 7 CIP(+) samples and 6 CIP(-) samples can be accessed in the Genome Sequence Archive (GSA)-Human database under accession code HRA002094 and the processed scRNA-seq data required to reproduce the analysis and figures have been deposited on OMIX website with accession ID OMIX001006 and OMIX004420.The bulk RNA-sequencing dataset of 8 CIP lung tissues and 29 normal controls [27] has been deposited at https://data.mendeley.com/datasets/8c3x28r5hk/. All the data generated and materials used in this manuscript are available upon request.
