# Supplementary material for: Deciphering pathogenic cellular module at single-cell resolution in checkpoint inhibitor-related pneumonitis
Source: Oncogene. 2023 Aug 31;42(42):3098–112. doi: 10.1038/s41388-023-02805-4 (PMC10575783; doi:10.1038/s41388-023-02805-4)
Supplement: Supplementary file 1 — supplementary figures and tables legends [file 41388_2023_2805_MOESM1_ESM.docx]

**Supplemental Material Legends:**

Supplemental Fig. 1, scRNA-seq data analysis. A, Distribution of sample pathology in 76,496 high-quality BALF cells. B, Distribution of number of genes and UMIs detected per cell after filtering in CIP(+) and CIP(-) groups. C,D, Scatter plot showing the linear correlation between the number of genes and the number of UMIs detected in HSPCs. Colors indicate groups (left) and cell types (right). E, number of genes and UMIs detected per cell type after filtering. F, Heatmap showing averaged expression of top 8 marker genes.

Supplemental Fig. 2, T cell characteristics. A, Sub-clustering of all T cells revealed 12 cell-subclusters. B, Heat map showing the scaled expression of top 7 differentially expressed genes among 12 T cell-subclusters. C, The expression level of T cell functional scores. D, The proportion of sample contributions per annotated T cell-subclusters. E, Heatmap showing the transcriptome similarities among each T cell subset. F, The proportion of sample contributions per annotated T cell clone subtypes.

Supplemental Fig. 3, Myeloid cell characteristics and cytokine assay validation. A, Heat map showing the scaled expression of top 5 differentially expressed genes among 9 myeloid cell-subclusters. B, Heat map showing the scaled expression of enriched pathway among 9 myeloid cell-subclusters. C, Functional enrichment analysis showing significant hallmark gene sets enriched in CIP(+) associated myeloid cell clusters compared with CIP(-) associated myeloid cell clusters. NES normalized enrichment score. D,E, The expression of inflammatory cytokines and receptors in BALF of CIP(+) and CIP(-) group.

Supplemental Fig. 4, Trajectory analysis of myeloid cell subclusters. A,B, Monocle2 showing the differentiation trajectory among myeloid cell subclusters. C, Heatmap showing dynamic changes in gene expression along the pseudotime of myeloid cell subclusters differentiation trajectory.

Supplemental Fig. 5, Person correlation coefficient of cell types abundance.

Supplemental Fig. 6, A, Barplot showing the number of ligand-receptor number per cell type. B, Circle plots showing the significant ligand-receptor pairs between CXCL13+ T cells and LAMP3-DC.

Supplementary Table 1, Summary of clinical characteristics, CIP onset, and treatment history of patient groups included in scRNA-seq analysis, Related to Fig. 1b.

Supplementary Table 2, CIP treatment history for each patient included in scRNA-seq studies, Related to Fig. 1b.

Supplementary Table 3, Characteristics of CIP development and clinical management for each patient included in scRNA-seq studies, Related to Fig. 1b.

Supplementary Table 4, DEGs of 12 main cell types, related to figure 1d

Supplementary Table 5, Clinical characteristics of each patient included in flow cytometry analysis, related to Fig. 1g.

Supplementary Table 6, Summary of clinical characteristics, CIP onset, and treatment history of patient groups included in flow cytometry analysis, related to Fig. 1g.

Supplementary Table 7, DEGs of CD8 T subclusters, related to figure 2b

Supplementary Table 8, DEGs of CD4 T subclusters, related to figure 4b

Supplementary Table 9, DEGs of myeloid cells subclusters, related to figure 6c and Supplemental figure S3a

Supplementary Table 10, myeloid cells signature, related to Figure. 6e

Supplementary Table 11, Cytokine assay for examination of BALF in CIP(+) and CIP(-) samples, related to Supplementary Fig. 3d, e
