# Supplementary figures and images for: Deciphering pathogenic cellular module at single-cell resolution in checkpoint inhibitor-related pneumonitis
Source: Oncogene. 2023 Aug 31;42(42):3098–112. doi: 10.1038/s41388-023-02805-4 (PMC10575783; doi:10.1038/s41388-023-02805-4)

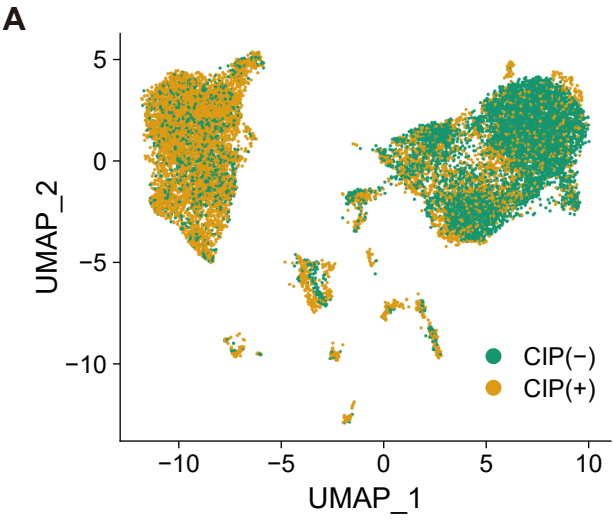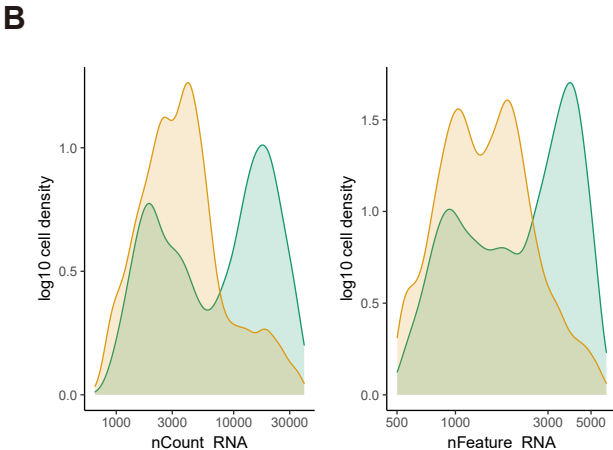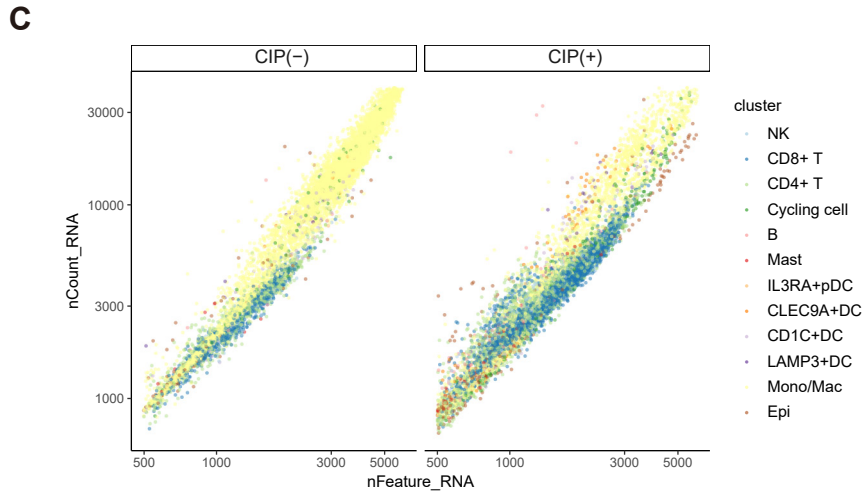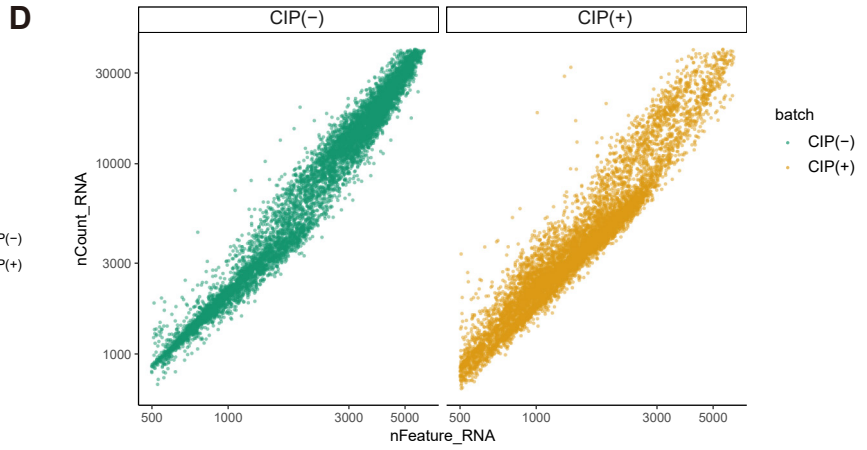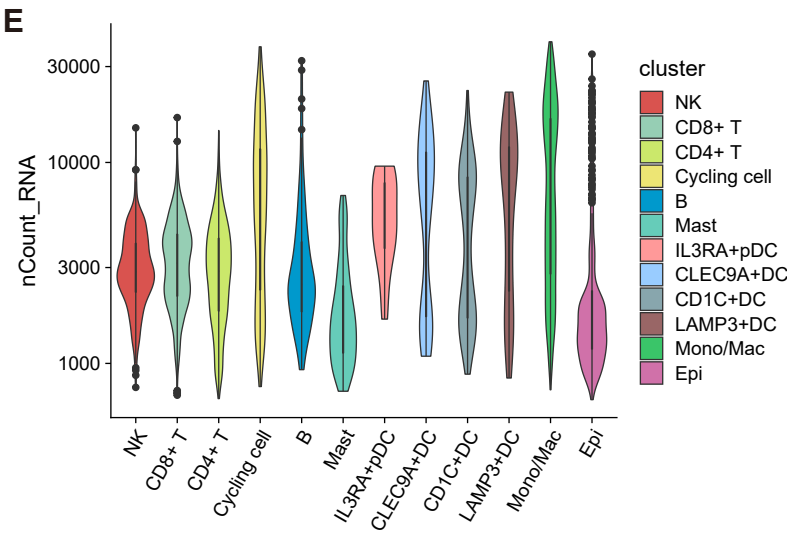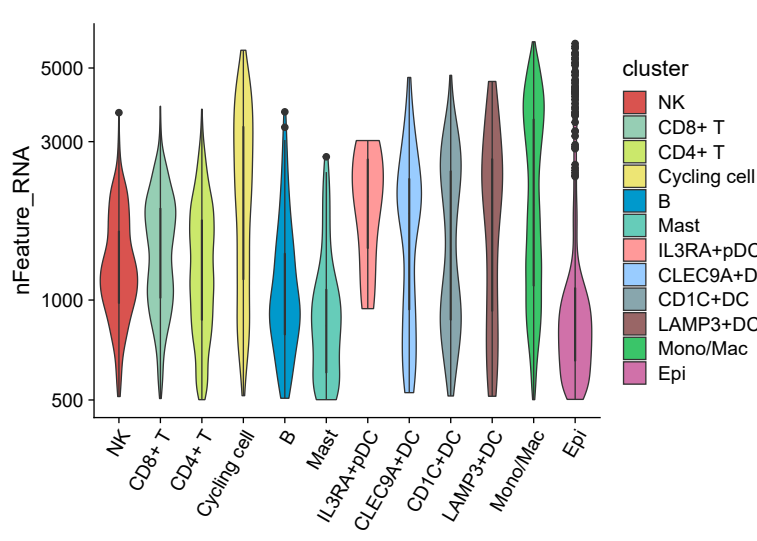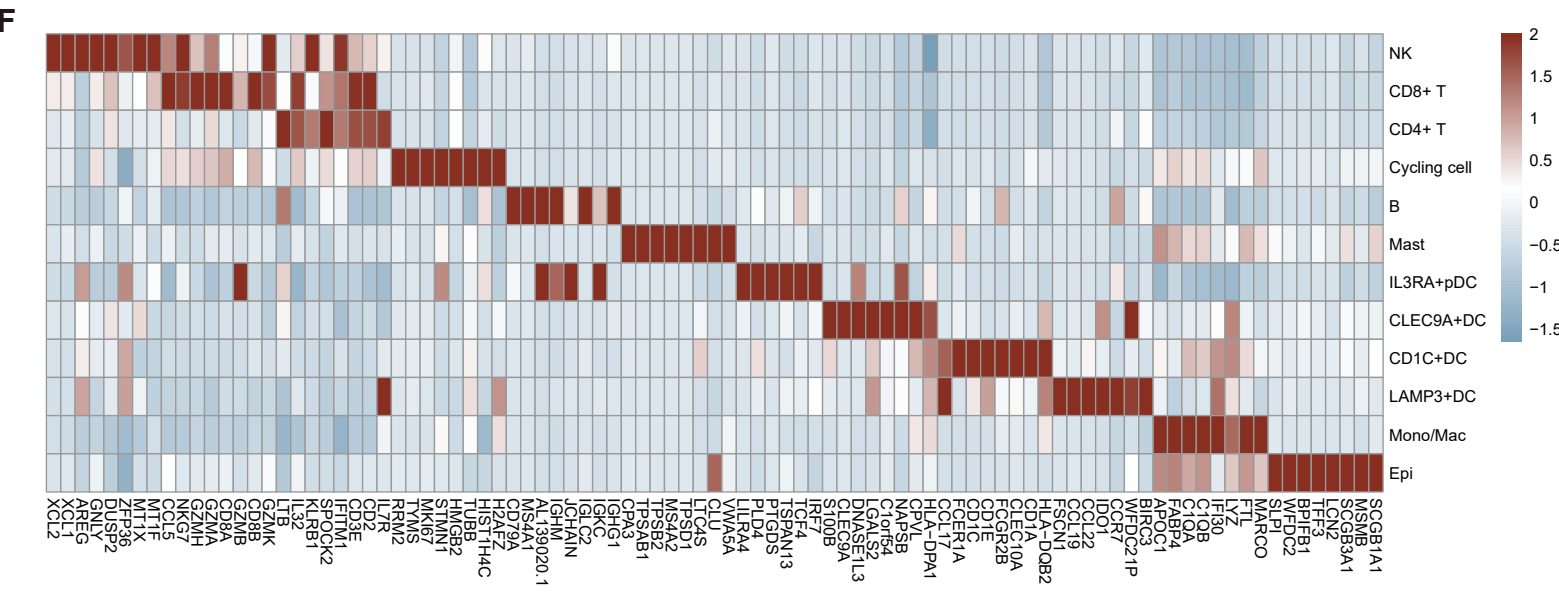

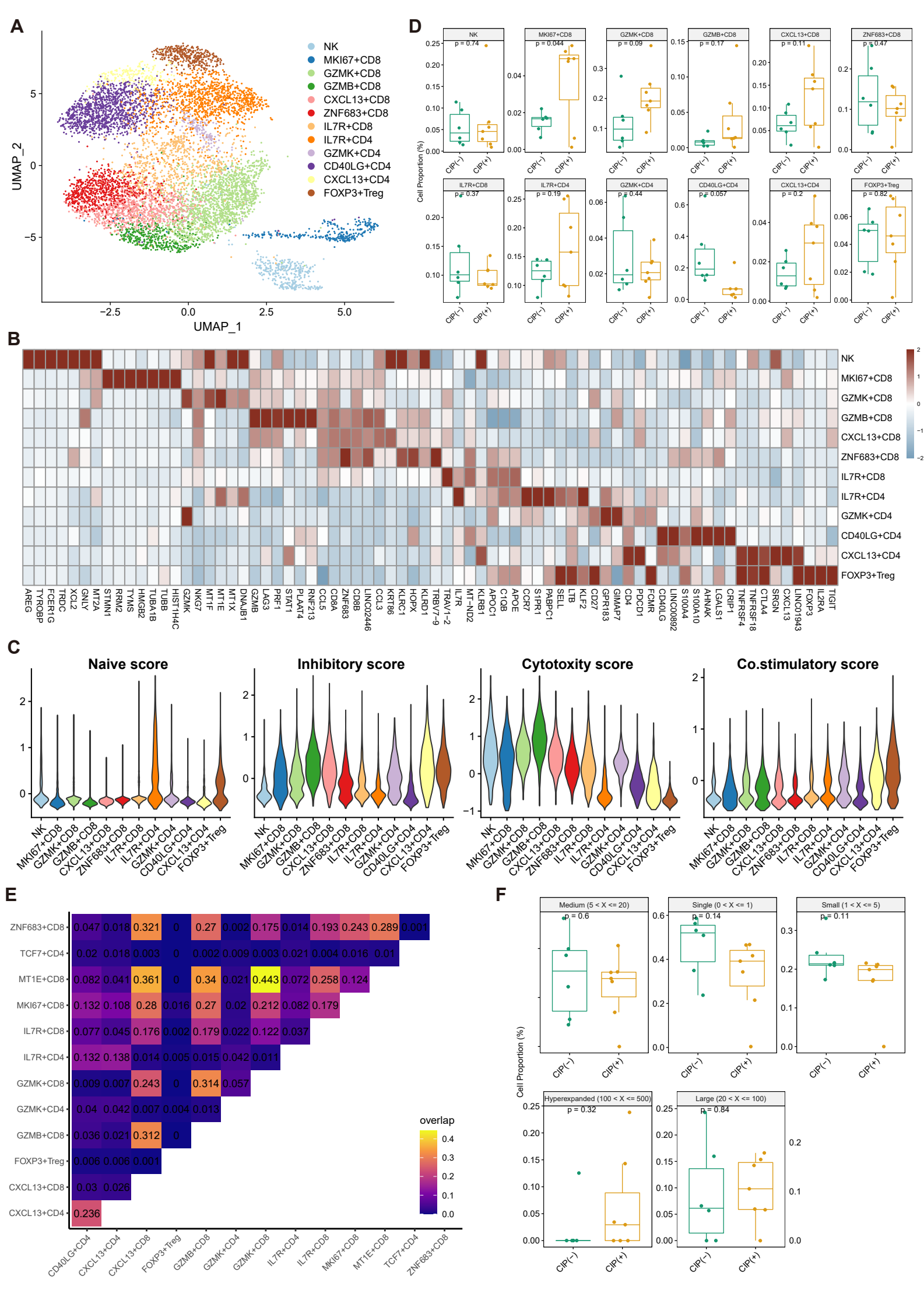

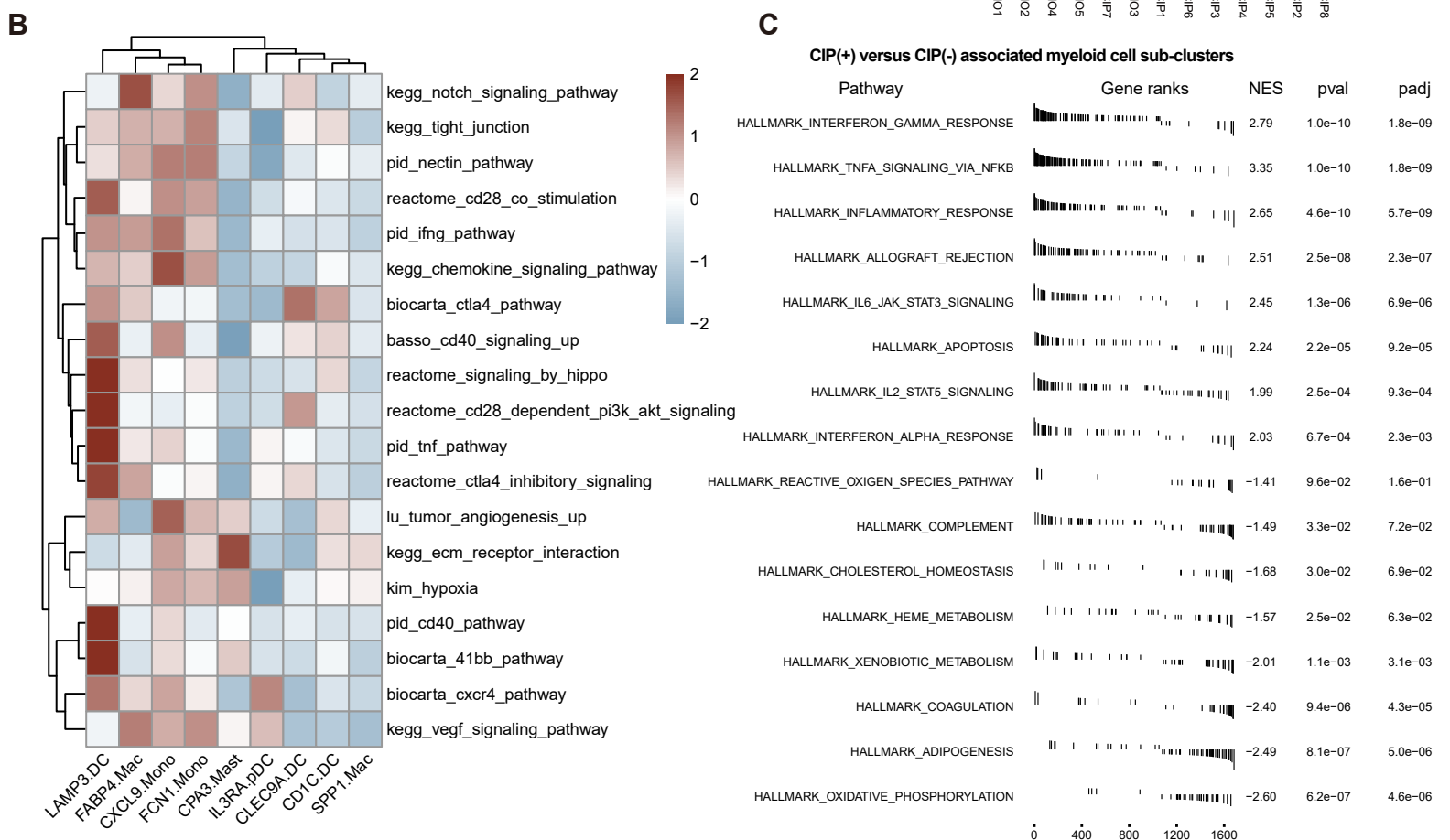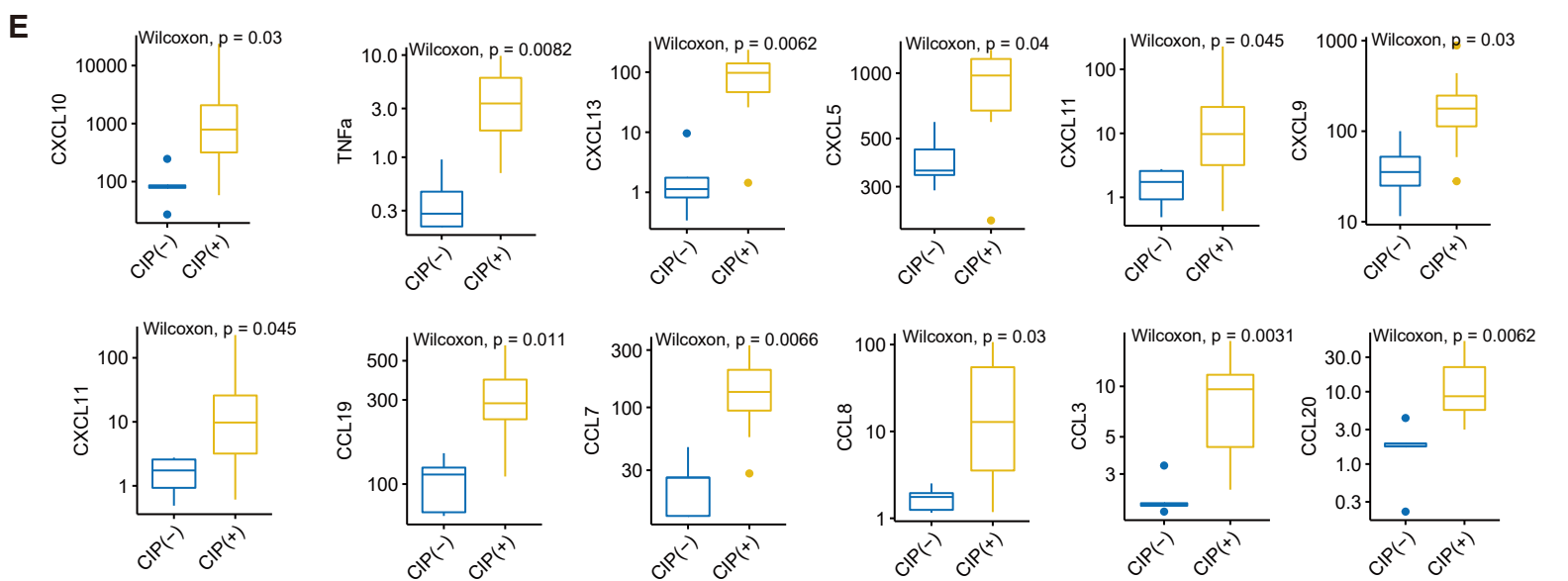

**A**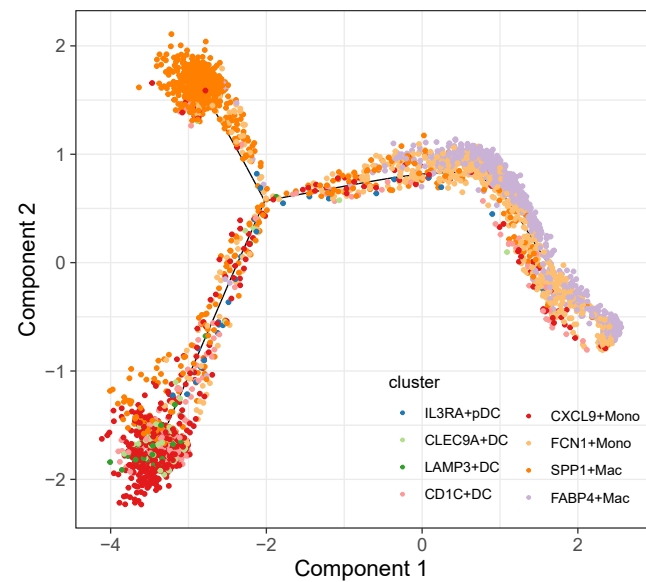**B**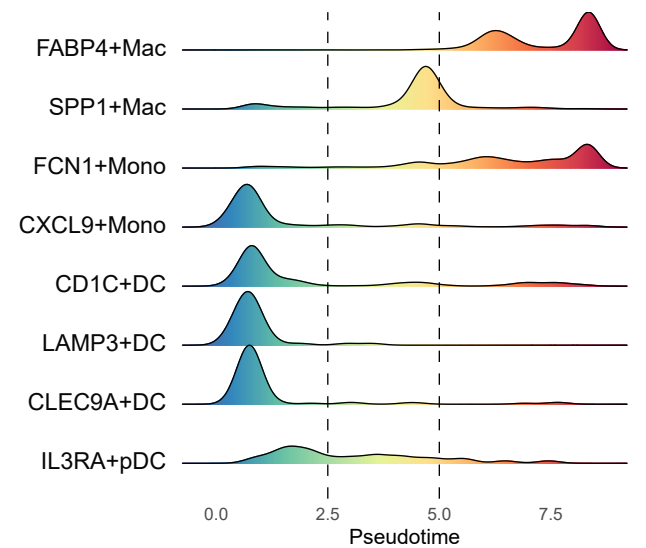**C**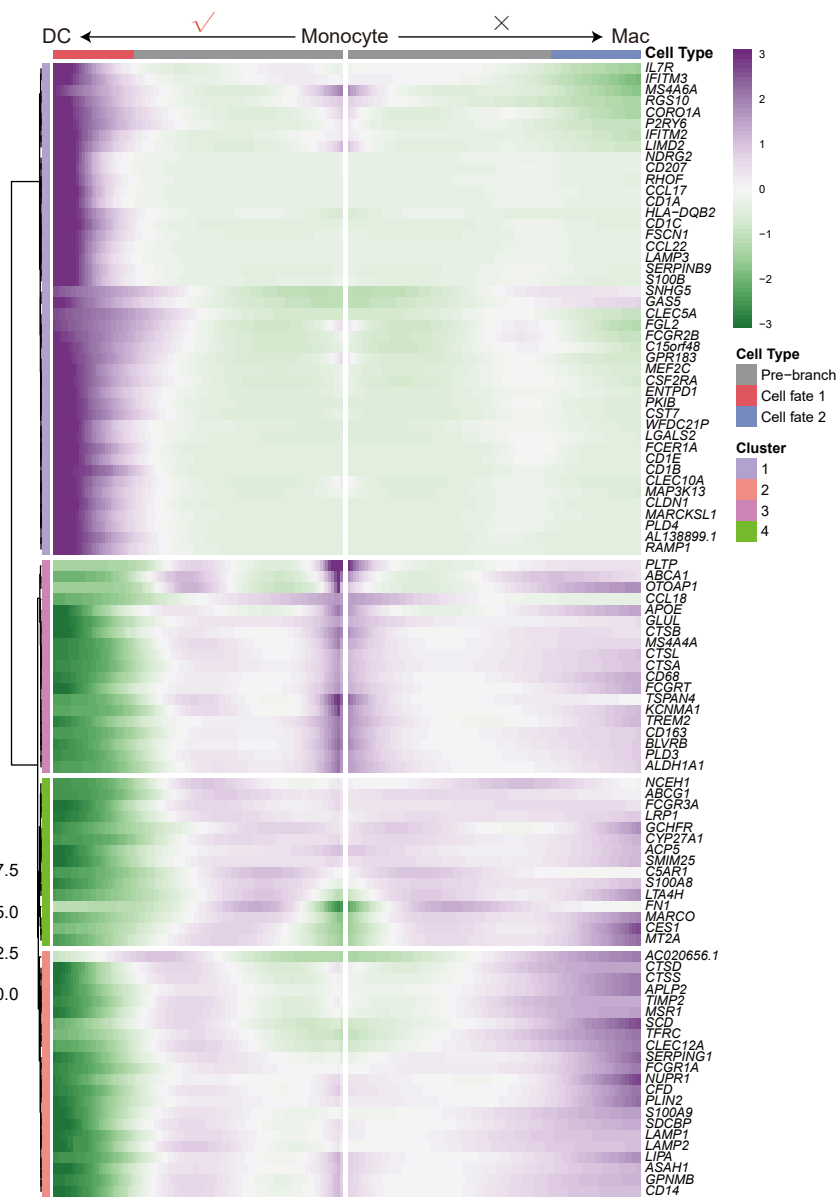

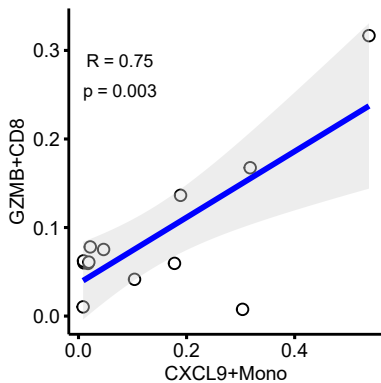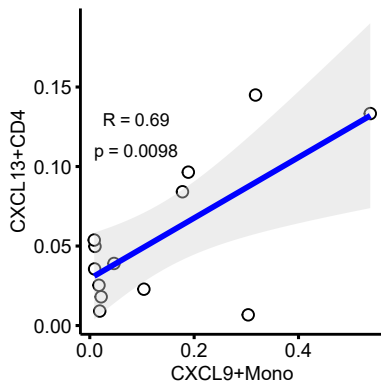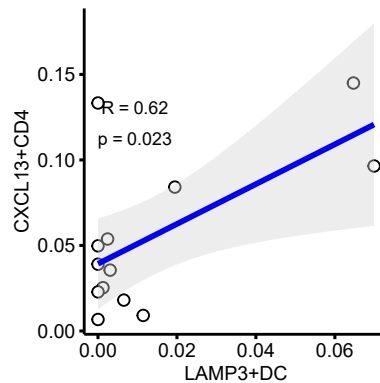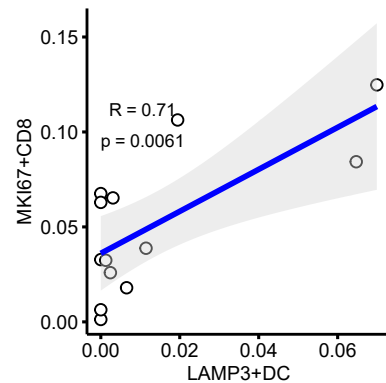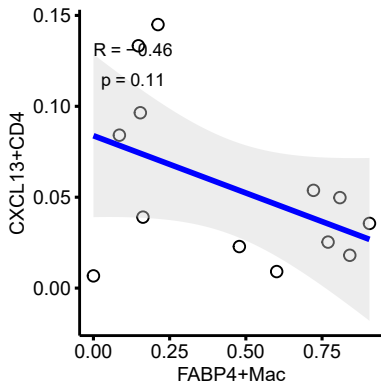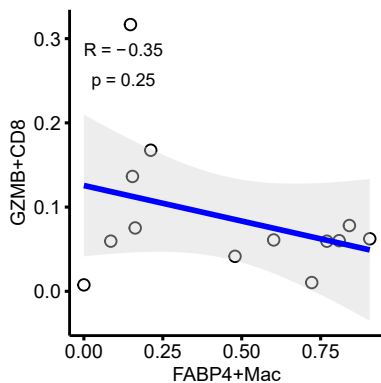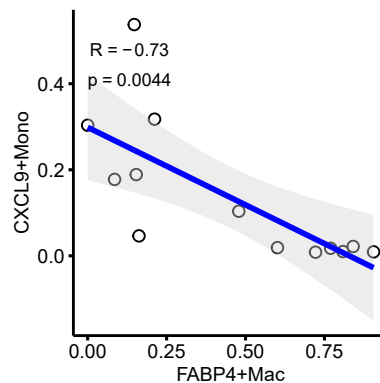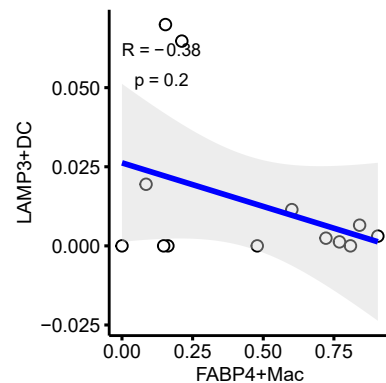

A

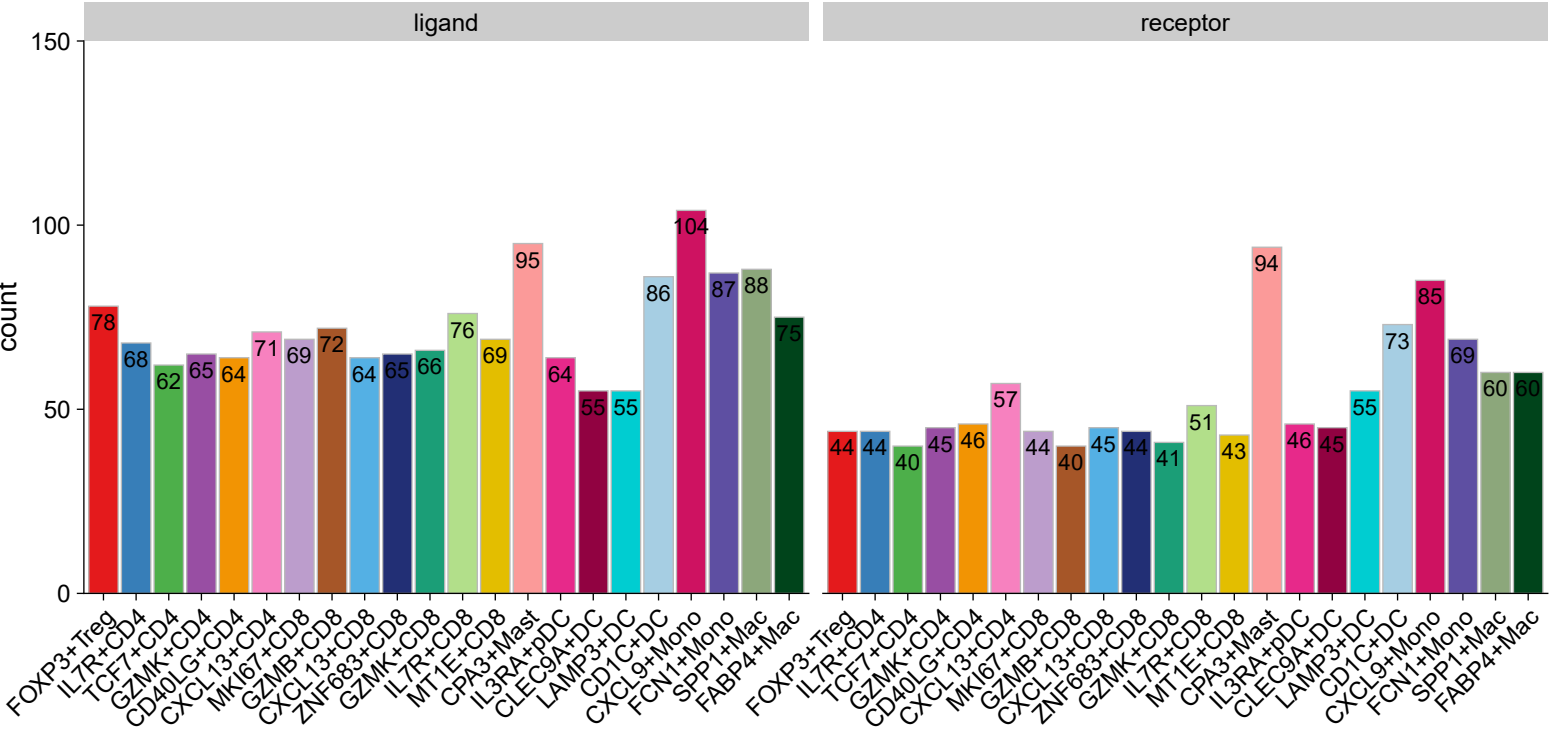

B

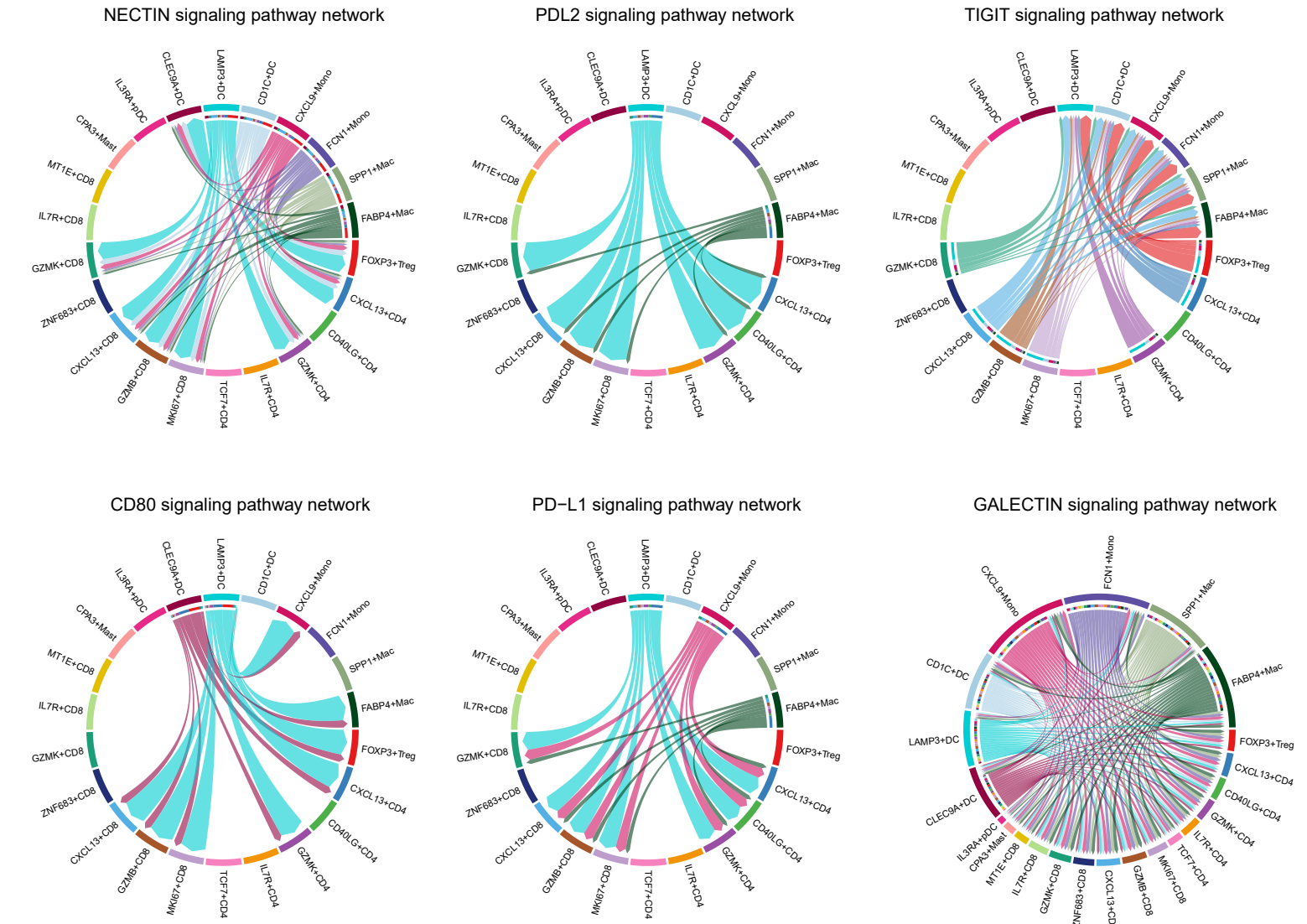

Supplement: Supplementary file 2 — Supplementary figures [file 41388_2023_2805_MOESM2_ESM.pdf]
